# Analyse von Einflussfaktoren auf ambulante pädiatrische Antibiotikaverordnungen in Bielefeld 2015–2018

**DOI:** 10.1007/s00103-024-03891-9

**Published:** 2024-06-05

**Authors:** Reinhard Bornemann, Andreas Heidenreich, Annika Hoyer, Amir Mohsenpour, Roland Tillmann

**Affiliations:** 1 AG 2 Bevölkerungsmedizin und Versorgungsforschung, Fakultät für Gesundheitswissenschaften, Universität Bielefeld, Universitätsstraße 25, 33615 Bielefeld, Deutschland; 2 Institut für Sozialmedizin und Epidemiologie, Universität zu Lübeck, Lübeck, Deutschland; 3 Biostatistik und Medizinische Biometrie, Medizinische Fakultät OWL, Universität Bielefeld, Bielefeld, Deutschland; 4 AG 2 Bevölkerungsmedizin und Versorgungsforschung, Fakultät für Gesundheitswissenschaften, Universität Bielefeld, Bielefeld, Deutschland; 5 Praxis für Kinder- und Jugendmedizin Roland Tillmann, Bielefeld, Deutschland; 6 Ärztenetz Bielefeld, Bielefeld, Deutschland

**Keywords:** Antibiotic Stewardship, Arzneimittelpatienten, Ambulante Versorgung, Verordnungsvarianz, Kinder- und Jugendmedizin, Antimicrobial stewardship, Medication patients, Ambulatory care, Prescription variance, Paediatrics

## Abstract

**Hintergrund:**

Beim Antibiotika-Verordnungsverhalten bestehen deutliche regionale Unterschiede. Die Ursachen dafür sind noch weitgehend unklar. Neben demografischen und morbiditätsbezogenen spielen auch arztindividuelle bzw. „kulturelle“ Faktoren eine Rolle. Um diese besser einordnen zu können, ist eine differenzierte Analyse unter Einbezug von Diagnosedaten erforderlich.

**Methoden:**

Es erfolgte eine Sekundärdatenanalyse der über die Kassenärztliche Vereinigung Westfalen-Lippe (KVWL) verfügbaren Infektionsdiagnosen bzw. Antibiotikaverordnungen von ambulant tätigen Kinderärztinnen und -ärzten im KV-Bezirk Bielefeld der Jahre 2015–2018. Zusätzlich erfolgten algorithmisierte 1:1-Verknüpfungen von Diagnosen und Verordnungen.

**Ergebnisse:**

Ausgewertet wurden 28.248 Verordnungen bei 262.969 Arzneimittelpatienten (AMP) sowie 90.044 Diagnosen, mit 11.131 1:1-Verknüpfungen. Circa 40 % der Verordnungen konnten somit einer Diagnose zugeordnet werden. Mit Blick auf das Verordnungsverhalten einzelner Praxen fanden sich, adjustiert auf die Nennergröße AMP, trotz vergleichbarer Alters- und Geschlechtsstruktur, z. T. deutliche Unterschiede. Dies betraf sowohl die Verordnungshäufigkeit als auch die Zusammensetzung der verordneten Substanzgruppen.

**Diskussion:**

Die gefundenen Varianzen im Verordnungsverhalten auf Praxisebene sind weder durch die demografische Zusammensetzung noch durch unterschiedliche Morbiditäten der jeweiligen Klientel hinreichend erklärbar. Individuelle Einstellungen bzw. lokale Verordnungskulturen dürften eine relevante Rolle spielen. Hierin liegt ein wichtiger Ansatz für Antibiotic Stewardship (ABS). Die dargelegte Methodik bietet sich über das vorgestellte Gebiet der ambulanten Pädiatrie hinaus als Modell für die detailliertere Analyse auch in anderen ambulanten Fachgruppen an.

**Zusatzmaterial online:**

Zusätzliche Informationen sind in der Online-Version dieses Artikels (10.1007/s00103-024-03891-9) enthalten.

## Abstract (en)

**Background:**

There are significant regional differences in antibiotic prescribing behaviour. The reasons for this are still largely unknown. Beneath demographic and morbidity-related factors, doctor-specific or “cultural” factors may also play a role. A differentiated analysis including diagnostic data is needed to put these data into context.

**Methods:**

A data analysis with secondary data available via the Westphalia-Lippe Association of Statutory Health Insurance Physicians (KVWL) was conducted on infection diagnoses and antibiotic prescriptions of outpatient paediatricians in the KV district of Bielefeld from 2015 to 2018. In addition, algorithmized 1:1 connections between diagnoses and prescriptions were performed.

**Results:**

For 262,969 “medication patients” (AMP), 28,248 antibiotic prescriptions and 90,044 infection diagnoses were evaluated, from which 11,131 1:1 connections could be generated. Concerning the prescribing behaviour of individual paediatric GP offices, after adjusting for the denominator AMP and despite a comparable age and gender structure, there were some significant differences. This affected both the frequency of prescriptions and the qualitative composition of the substance groups prescribed.

**Discussion:**

The differences in antibiotic prescribing behaviour, even at GP office level, cannot be adequately explained by the demographic composition or different morbidities of the respective clientele. Individual attitudes and local prescribing cultures are likely to play a relevant role. To address these offers an important approach for antibiotic stewardship (ABS). In addition to the area of outpatient paediatrics presented here, the methodology described can also be used as a model for more detailed analysis in other outpatient speciality groups.

## Hintergrund

Bakterielle Resistenzen gegen Antibiotika (AB) stellen ein zunehmendes Problem in der Medizin dar. Ein unangemessenes AB-Verordnungsverhalten trägt maßgeblich hierzu bei. Maßnahmen zur Förderung eines rationalen Verordnungsverhaltens werden unter dem Begriff „Antibiotic Stewardship“ (ABS) zusammengefasst. Ein Hauptaugenmerk von ABS liegt auf der Beobachtung und Analyse des Verordnungsverhaltens sowohl auf räumlicher Ebene als auch innerhalb verschiedener ärztlicher Fachgruppen (FG). Hier sind jeweils große Varianzen festzustellen, mit entsprechendem Verbesserungspotenzial. Solche Varianzen finden sich auf innereuropäischer [1] und nationaler Ebene [2]. Ebenso existieren bereits Daten für einzelne FG auf geografischer Bezugsebene [3].

Es wird angenommen, dass diese Varianzen nur zum Teil durch unterschiedliche Patientencharakteristika bzw. Morbiditäten zu erklären sind und daneben individuelle Verhaltensunterschiede wie auch lokale „Verordnungskulturen“ eine relevante Rolle spielen [4]. Mit Blick hierauf wurde 2016 von Bielefelder Kinder- und Jugendärztinnen und -ärzten das Projekt „Antibiotische Therapie in Bielefeld“ (AnTiB, [5]) gegründet. Das primäre Projektziel war, mittels Kommunikation innerhalb der FG eine Angleichung des Verordnungsverhaltens und damit die Verordnungssicherheit zu steigern und Konflikte zu reduzieren [6]. Ein wesentlicher Baustein des Projektes war die Entwicklung von pädiatrischen AB-Verordnungsempfehlungen für den ambulanten Bereich, die formal am 01.01.2017 für die FG Pädiatrie in Bielefeld in Kraft gesetzt wurden und inzwischen auch in adaptierter Form bundesweit genutzt werden [7].

2022 erfolgte eine erste Auswertung der auch dieser Arbeit zugrunde liegenden Daten [3]. Die vorliegende Arbeit erweitert die Datenanalyse und bezieht neben den Verordnungsdaten die im gleichen Zeitraum gestellten Diagnosecodierungen mit ein, um den Faktor Morbidität näher eingrenzen zu können. Dies ist allerdings schwierig, da die Verordnungsdaten taggenau, die Diagnosedaten hingegen nur quartalsbezogen verfügbar sind. Mit dieser Problematik hat sich erstmals eine Arbeit befasst [8], an deren Methodik hier angeknüpft wird. Auch sollte untersucht werden, inwieweit die Implementierung des AnTiB-Projektes einen Einfluss auf das lokale AB-Verordnungsverhalten gehabt haben könnte.

Die Fragestellungen der vorliegenden Untersuchung lauten:

1. Wie können ambulante Antibiotikaverordnungsdaten gewonnen und im Sinne von ABS differenziert und gezielt ausgewertet werden?
2. In welchem Umfang und mit welchen Varianzen werden von ambulant tätigen Kinder- und Jugendärzt:innen Antibiotika verordnet, sowohl insgesamt als auch unterteilt nach einzelnen Wirkstoffgruppen?
3. Welche Einflussfaktoren auf Antibiotikaverordnungen lassen sich seitens der Patient:innen bzw. der verordnenden Ärzt:innen erkennen? Welche Rolle spielen die den Patient:innen zugewiesenen Infektionsdiagnosen?
4. Welchen Einfluss hat der zeitliche Verlauf auf die Antibiotikaverordnungen? Lassen sich dabei Effekte des Projekts AnTiB erkennen?

## Methodik

### Ausgangsdatensätze

Zur Datenbasis wird zunächst auf die Vorgängerarbeit verwiesen [3]. Kurzgefasst wurden untersucht: die AB-Verordnungen bei GKV-versicherten Kindern bis 18 Jahre durch ambulant tätige Kinder- und Jugendärzt:innen der KV-Bezirksstelle Bielefeld in den Jahren 2015–2018. Während in [3] eine deskriptive Auswertung stattfand, erfolgte in der vorliegenden Arbeit eine tiefergehende Analyse der Verordnungsdaten, unter Hinzunahme weiterer Datenquellen.

Einbezogen wurden dabei folgende 3 jeweils von der Kassenärztlichen Vereinigung Westfalen-Lippe (KVWL) übermittelte Datensätze:

*Verordnungen der AB zur systemischen Anwendung* (ATC^1^ J01, [9, 10]), wobei gilt:

- Jede einzelne Verordnung kann exakt einer individuellen Patientenidentifikationsnummer (Pat.-ID), einem individuellen Verordner (LANR^2^) sowie seiner Praxis (BSNR^3^) zugeordnet werden.
- Das Datum der Verordnung (resp. der Einlösung in der Apotheke) liegt taggenau vor.

*Arzneimittelpatienten* (AMP, [11]) als „Nennergröße“, da die arztindividuelle Verordnungshäufigkeit zunächst von der Anzahl der in der jeweiligen Praxis versorgten Patient:innen abhängt: als AMP zählt jede/r GKV-Patient:in, die/der ein Arzneimittel auf einem Kassenrezept verordnet bekommt und dieses in einer Apotheke einlöst.

- AMP-Daten können ebenfalls individuellen LANR und BSNR zugeordnet werden, Patientendaten hingegen sind nur aggregiert für Alter und Geschlecht verfügbar.
- AMP-Daten stehen monats-, quartals- und jahresweise zur Verfügung, wobei hier „Quartal“ gewählt wurde.

*Infektionsdiagnosen:* Bei der jeweils ersten Praxiskonsultation einer Patientin oder eines Patienten im Quartal wird mindestens eine Diagnose gemäß ICD-10 GM^4^ kodiert; bei weiteren Kontakten in demselben Quartal können Diagnosen hinzukommen. Jeder Diagnosecode erhält einen Zusatz zur Diagnosesicherheit: „G = gesichert“, „V = Verdacht auf“, „Z = Zustand nach“ und „A = Ausschluss von“.

- Bei den Diagnosedaten liegen die individuellen Pat.-ID vor, dazu jedoch nur die BSNR bzw. nicht die LANR.
- Diagnosedaten liegen nur quartalsbezogen vor.

Aus der Gesamtheit der Diagnosen wurden diejenigen identifiziert, bei denen grundsätzlich eine AB-Gabe denkbar erschien. Hierbei wurde zunächst die Vorgabe von [8] genutzt. Diese wurde um die unserer Ansicht nach ebenfalls zu berücksichtigenden ICD-Codes A49 und B99 (unspezifische Infektionen) sowie L01–03 und L08 (Hautinfektionen) ergänzt (siehe Tab. Z1 im Onlinematerial zu diesem Artikel; alle zusätzlichen Materialien sind mit „Z“ gekennzeichnet).

### Verknüpfung der Verordnungs- und der Diagnosedaten

Die methodische Herausforderung lag zunächst darin, die 3 genannten inhaltlich und zeitlich unterschiedlich aufgebauten Datenquellen miteinander zu verknüpfen.

Gemäß [8] wurden einzelne AB-Verordnungen entsprechenden Diagnosen so zugeordnet: Im Falle nur einer AB-Verordnung einer Ärztin oder eines Arztes bzw. einer Praxis im Quartal und ebenfalls nur einer von dieser Praxis gestellten relevanten Diagnose besteht eine hohe Wahrscheinlichkeit, dass diese Verordnung auf der besagten Diagnose basiert. Damit ließen sich 1:1-Verknüpfungen zwischen Verordnungen und Diagnosen generieren und auswerten. Wurden einem/er Patient:in in einem Quartal mehrere AB verordnet, war das zuerst verordnete AB für die Verknüpfung maßgeblich. Auf diese Weise ließen sich insgesamt 41.078 potenziell anschlussfähige Diagnosen identifizieren, denen 11.131 AB-Verschreibungen zugeordnet werden konnten.

### Statistische Analyse

Für die statistische Analyse der Anzahl an verordneten AB wurden Poisson-Regressionsmodelle mit zufälligem Effekt (Random Intercept Models, [12]) verwendet. Dabei werden Unterschiede zwischen den Praxen in Form eines unterschiedlichen Ausgangswerts in der AB-Verschreibungskultur gesehen. Dies dient zur Berücksichtigung des unterschiedlichen Verordnungsverhaltens der eingeschlossenen Betriebsstätten („Cluster-Effekt“) und der wiederholten Messungen über den Zeitraum der betrachteten Quartale. Der gesamte Beobachtungszeitraum 2015–2018 wird unterteilt in „Prä-AnTiB“ (Quartale Q1–Q4 in 2015/16) und „Post-AnTiB“ (Q1–Q4 in 2017/18), vor dem Hintergrund, dass per 01.01.2017 die neu erarbeiteten pädiatrischen AB-Verordnungsempfehlungen publiziert und innerhalb der lokalen Fachgruppe intensiv kommuniziert wurden.

Das erste Regressionsmodell beschreibt die zeitliche Entwicklung der verschriebenen AB unter Berücksichtigung der Anzahl an Arzneimittelpatient:innen pro Betriebsstätte, die als Offset eingeht. Weiterhin wird für das Geschlecht und Alter der Patient:innen adjustiert. Modelle 2 und 3 analysieren die Anzahl an verschriebenen AB unter Berücksichtigung der Anzahl an AB-relevanten Diagnosen pro Betriebsstätte, jeweils getrennt für den „Prä-“ bzw. „Post-AnTiB“-Zeitraum. Dabei wird zusätzlich für die Diagnosegruppe adjustiert.

Im Rahmen einer Sensitivitätsanalyse (Modell 4) wird die Anzahl der verordneten AB unter Berücksichtigung der Anzahl an AB-relevanten Diagnosen pro Betriebsstätte als Outcome betrachtet. Exposition ist dabei der jeweilige Verordnungszeitraum. Zusätzlich wird für die Diagnosegruppe adjustiert. Als Effektgrößen werden relative Risiken mit zugehörigen 95 %-Konfidenzintervallen (KI) angegeben. Die statistische Auswertung erfolgte mit der Software R (R Foundation for Statistical Computing, Wien, Österreich; Version 4.2.1).

## Ergebnisse

Im Zeitraum vom 01.01.2015 bis 31.12.2018 wurden die AB-Verordnungs- und Diagnosedaten von 28 ambulant tätigen Kinderärzt:innen, verteilt auf 16 Praxen (9 Einzel-, 4 Doppel-, 1 Dreier- und 2 Viererpraxen), im KV-Bezirk Bielefeld anhand von KV-Daten erhoben. Nach Datenbereinigung (Eingrenzung des Alters auf < 18 Jahre, Ausschluss bei fehlender Geschlechtsangabe) konnten 28.248 Verordnungsfälle einbezogen werden. Diese verteilten sich nach Quartalen gemäß Abb. Z1. Dabei ist zu beachten, dass 3 Praxen erst später hinzukamen: Praxis O im Q4 2015, N im Q1 2016 und J im Q1 2018. Alle anderen Praxen waren über den gesamten Zeitraum hinweg aktiv.

Die verfügbaren Diagnose-Rohdaten wurden entsprechend der Ein- bzw. Ausschlusskriterien auf die auszuwertende Größe von 90.044 reduziert (Abb. Z2, Diagnosedaten). In Bezug auf die AMP konnten nach Eingrenzung auf < 18 Jahre 262.969 Datensätze in die Auswertung einbezogen werden (Abb. Z3).

Die Aufschlüsselung der AB-Verordnungen sowie der AMP nach Altersgruppen bzw. nach Geschlecht in den einzelnen Praxen zeigt Tab. 1. Diese Verordnungen waren Teil von insgesamt 262.969 AMP. Dies entspricht einer Wahrscheinlichkeit von Verordnungen/AMP von 10,7 % über alle 16 Praxen, mit einer Spannweite von ca. 6–18 %, mit 2 Ausnahmen: Praxis C mit nur 1,9 % vs. Praxis O mit 25,0 %.

**Tab. 1:** Teilnehmende Praxen, deren Verordnungszahlen (*AMP*) und Antibiotikaverordnungen, aufgeschlüsselt nach Altersgruppen und Geschlecht

	Arzneimittelpatient:innen								Antibiotikaverordnungen								
	0 bis < 2	2 bis < 6	6 bis < 10	10 bis < 15	15 bis < 18	Gesamt	Weibl. (in %)	Männl. (in %)	0 bis < 2	2 bis < 6	6 bis < 10	10 bis < 15	15 bis < 18	Gesamt	Weibl. (in %)	Männl. (in %)	AB:AMP (in %)
*A*	3804	4998	3413	2545	670	15.430	49,1	50,9	410	970	543	420	146	2489	49,1	50,9	16,1
*B*	7111	8520	4434	3195	619	23.879	46,9	53,1	365	1165	512	247	72	2361	51,6	48,4	9,9
*C*	1993	3451	2015	1108	165	8732	50,4	49,6	20	64	47	23	13	167	60,9	39,1	1,9
*D*	7991	13.411	8649	7946	3342	41.339	46,0	54,0	225	1062	713	473	274	2747	48,0	52,0	6,6
*E*	3946	6628	4019	2981	797	18.371	47,9	52,1	130	574	357	261	81	1403	58,7	41,3	7,6
*F*	3884	5063	2713	1318	189	13.167	48,0	52,0	96	329	231	104	37	797	58,8	41,2	6,1
*G*	4614	8102	4729	3283	896	21.624	46,9	53,1	194	817	433	272	117	1833	50,0	50,0	8,5
*H*	1740	3102	1657	1181	313	7993	50,1	49,9	156	552	283	212	75	1278	55,1	44,9	16,0
*I*	2242	3722	2644	1971	507	11.086	50,0	50,0	173	775	513	287	110	1858	49,2	50,8	16,8
*J*	625	876	509	298	91	2399	47,1	52,9	24	145	90	39	16	314	52,8	47,2	13,1
*K*	2211	3604	1895	1402	383	9495	49,8	50,2	87	359	176	121	30	773	59,2	40,8	8,1
*L*	1753	2791	1437	975	240	7196	45,7	54,3	47	212	121	81	25	486	48,9	51,1	6,8
*M*	6108	9710	6751	5476	1400	29.445	48,0	52,0	476	1532	947	599	254	3808	49,9	50,1	12,9
*N*	2646	3875	2448	1618	307	10.894	50,1	49,9	285	878	463	281	91	1998	51,3	48,7	18,3
*O*	2620	5860	4370	3854	1155	17.859	48,1	51,9	506	1922	1098	697	242	4465	47,9	52,1	25,0
*P*	6439	8322	4850	3469	980	24.060	46,7	53,3	165	604	373	248	81	1471	50,5	49,5	6,1

*A, B, C usw.* teilnehmende Praxen, *„0 bis <* *2“* *ff.* Patientenalter in Jahren, *AB:AMP* Anteil der AB-Verordnungen am Gesamt der Arzneimittelpatient:innen

Wird lediglich die Altersgruppe 15 bis < 18 jeweils auf Praxisebene betrachtet, so zeigt sich einerseits ein weitgehend ausgeglichenes Geschlechterverhältnis bei den AMP (51,7 % m vs. 48,3 % w), hingegen ein Überwiegen der AB-Verordnungen bei den Mädchen (42,9 % m vs. 57,1 % w; Tab. Z2). Mit Blick auf die jeweiligen Anteile von AB-Verordnungen an AMP nach Quartalen zeigen sich jeweils deutlich höhere Anteile in den Winter- und niedrigere in den Sommerquartalen (Abb. Z4). Das Verhältnis von AMP und AB-Verordnungen ist praxisindividuell sehr heterogen (Abb. Z5).

Die verordneten AB wurden gruppiert in: Betalactam-AB, Penicilline (J01C…) mit der Unterfraktion „Schmalspektrum“ (ATC J01CE01–J01CE10 und J01CF01–J01CF06), Andere Betalactam-AB (J01D…), Sulfonamide und Trimethoprim (J01E…), Makrolide, Lincosamide und Streptogramine (J01F…), Sonstige (J01A…, J01G…, J01M…, J01X…), und ihr jeweiliger Anteil pro Praxis über den Erhebungszeitraum 2015–2018 dargestellt (Tab. 2). Hierbei fällt auf:

- Penicilline bilden die am häufigsten verordnete Substanzgruppe, mit überwiegend ca. 50–75 %, von Praxis A mit 9,3 % bis Praxis L mit 75,5 %. Kommen die anderen Betalactam-AB hinzu, liegen die Betalactame insgesamt bei ca. 80–90 %; auch hier liegt Praxis A mit nur 28,9 % deutlich darunter.
- Bei den Makroliden liegen fast alle Praxen im einstelligen Bereich, Praxis A mit 62,8 % hingegen weit darüber.
- Bei den Sulfonamiden und Trimethoprim ist die Spannweite deutlich geringer, mit ca. 5–10 %, von Praxis I mit 1,1 % bis Praxis F mit 15,2 %.
- Die Verordnungen der sonstigen AB bewegen sich sämtlich im niedrigen einstelligen Prozentbereich.

**Tab. 2:** Anteile der pro Praxis verordneten Antibiotika nach Substanzgruppen

	Penicilline	… hiervon Schmalspektrum*	Andere Betalactam-Antibiotika	Makrolide, Lincosamide und Streptogramine	Sulfonamide und Trimethoprim	Sonstige	Gesamt
*A*	232 (9,3 %)	20 (0,8 %)	488 (19,6 %)	1563 (62,8 %)	133 (5,3 %)	73 (2,9 %)	2489
*B*	1776 (75,2 %)	379 (16,1 %)	250 (10,6 %)	88 (3,7 %)	192 (8,1 %)	55 (2,3 %)	2361
*C*	60 (35,9 %)	4 (2,4 %)	37 (22,2 %)	60 (35,9 %)	8 (4,8 %)	2 (1,2 %)	167
*D*	1489 (54,2 %)	272 (9,9 %)	737 (26,8 %)	160 (5,8 %)	225 (8,2 %)	136 (5,0 %)	2747
*E*	875 (62,4 %)	286 (20,4 %)	265 (18,9 %)	128 (9,1 %)	119 (8,5 %)	16 (1,1 %)	1403
*F*	478 (60,0 %)	138 (17,3 %)	163 (20,5 %)	22 (2,8 %)	121 (15,2 %)	13 (1,6 %)	797
*G*	963 (52,5 %)	275 (15,0 %)	595 (32,5 %)	188 (10,3 %)	66 (3,6 %)	21 (1,1 %)	1833
*H*	941 (73,6 %)	387 (30,3 %)	194 (15,2 %)	43 (3,4 %)	85 (6,7 %)	15 (1,2 %)	1278
*I*	976 (52,5 %)	171 (9,2 %)	681 (36,7 %)	95 (5,1 %)	20 (1,1 %)	86 (4,6 %)	1858
*J*	177 (56,4 %)	64 (20,4 %)	93 (29,6 %)	22 (7,0 %)	22 (7,0 %)	0 (0 %)	314
*K*	459 (59,4 %)	125 (16,2 %)	201 (26,0 %)	17 (2,2 %)	87 (11,3 %)	9 (1,2 %)	773
*L*	367 (75,5 %)	164 (33,7 %)	52 (10,7 %)	34 (7,0 %)	29 (6,0 %)	4 (0,8 %)	486
*M*	2379 (62,5 %)	581 (15,3 %)	753 (19,8 %)	298 (7,8 %)	198 (5,2 %)	180 (4,7 %)	3808
*N*	1396 (69,9 %)	517 (25,9 %)	460 (23,0 %)	24 (1,2 %)	117 (5,9 %)	1 (0,1 %)	1998
*O*	2135 (47,8 %)	810 (18,1 %)	1830 (41,0 %)	467 (10,5 %)	19 (0,4 %)	14 (0,3 %)	4465
*P*	800 (54,4 %)	265 (18,0 %)	464 (31,5 %)	73 (5,0 %)	82 (5,6 %)	52 (3,5 %)	1471
*Md* *IQR*	*57,9* *%* *(52,5–64,4)*	*16,8* *%* *(13,7–20,4)*	*22,6* *%* *(19,4–30,1)*	*6,4* *%* *(3,6–9,4)*	*6,0* *%* *(5,1–8,1)*	*1,2* *%* *(1,0–3,1)*	–

*Md* Median, *IQR* Interquartilabstand

*ATC J01CE01–J01CE10 und J01CF01–J01CF06

(Die Praxis C wurde aufgrund ihrer geringen Verordnungszahl von 167 dabei jeweils ausgeklammert.)

Werden die Spektren der Infektionsdiagnosen in den jeweiligen Praxen (Tab. 3) betrachtet, liegen die Atemwegsinfekte an erster Stelle, überwiegend in der Spannbreite von ca. 60–80 %, von Praxis C mit 33,6 % bis Praxis L mit 91,2 %. Die Unterfraktion „Pneumonie“ (ICD J13, J14, J15, J16 und J18) rangiert zwischen 0,4 % bei Praxis C bis 7,6 % bei Praxis O. Die Otitis liegt überwiegend bei ca. 15–25 %, mit Minimum bei Praxis L mit 4,8 % und Maximum bei Praxis C mit 27,9 %. Hauterkrankungen bewegen sich im niedrigen einstelligen Bereich. Während die Rubrik „nicht näher bezeichnete bakterielle Infektionen“ überwiegend nur marginal angegeben wird, ragt hier Praxis A mit 9,2 % heraus. Diagnosen aus dem Urogenitalsystem weisen nur einen niedrigen einstelligen Prozentbereich auf, ähnlich beim Scharlach, am höchsten hier Praxis M mit 6,1 %. Sehr breit ist das Feld bei den „sonstigen Infektionskrankheiten“ von Einzelfällen bis 26,5 % bei Praxis C.

**Tab. 3:** Antibiotikarelevante Infektionskrankheiten nach Gruppe pro Praxis

	Atmungssystem	… hiervon Pneumonie*	Otitis	Haut	Bakterielle Inf., n. n. b.	Urogenitalsystem	Scharlach	Sonst. Inf.-krankheit	Gesamt
*A*	4802 (65,2 %)	114 (1,5 %)	1753 (23,8 %)	115 (1,6 %)	676 (9,2 %)	10 (0,1 %)	3 (0,0 %)	1 (0,0 %)	7360
*B*	5475 (73,2 %)	281 (3,8 %)	1700 (22,7 %)	128 (1,7 %)	12 (0,2 %)	3 (0,0 %)	117 (1,6 %)	42 (0,6 %)	7477
*C*	379 (33,6 %)	4 (0,4 %)	314 (27,9 %)	51 (4,5 %)	1 (0,1 %)	83 (7,4 %)	0 (0 %)	299 (26,5 %)	1127
*D*	3642 (63,4 %)	220 (3,8 %)	1552 (27,0 %)	348 (6,1 %)	6 (0,1 %)	23 (0,4 %)	147 (2,6 %)	26 (0,5 %)	5744
*E*	3536 (70,9 %)	86 (1,7 %)	899 (18,0 %)	95 (1,9 %)	144 (2,9 %)	108 (2,2 %)	121 (2,4 %)	85 (1,7 %)	4988
*F*	1520 (62,7 %)	92 (3,8 %)	636 (26,2 %)	72 (3,0 %)	1 (0,0 %)	81 (3,3 %)	91 (3,8 %)	25 (1,0 %)	2426
*G*	5188 (49,5 %)	374 (3,6 %)	2877 (27,4 %)	299 (2,9 %)	30 (0,3 %)	53 (0,5 %)	159 (1,5 %)	1884 (18,0 %)	10.490
*H*	3917 (70,2 %)	101 (1,8 %)	901 (16,1 %)	67 (1,2 %)	150 (2,7 %)	272 (4,9 %)	54 (1,0 %)	222 (4,0 %)	5583
*I*	5881 (79,9 %)	204 (2,8 %)	1213 (16,5 %)	130 (1,8 %)	16 (0,2 %)	25 (0,3 %)	95 (1,3 %)	1 (0,0 %)	7361
*J*	365 (60,4 %)	35 (5,8 %)	150 (24,8 %)	19 (3,1 %)	0 (0 %)	8 (1,3 %)	24 (4,0 %)	38 (6,3 %)	604
*K*	2045 (66,9 %)	38 (1,2 %)	476 (15,6 %)	65 (2,1 %)	3 (0,1 %)	11 (0,4 %)	26 (0,9 %)	432 (14,1 %)	3058
*L*	1974 (91,1 %)	54 (2,5 %)	104 (4,8 %)	19 (0,9 %)	1 (0,0 %)	45 (2,1 %)	23 (1,1 %)	0 (0 %)	2166
*M*	6456 (68,2 %)	348 (3,7 %)	1909 (20,2 %)	291 (3,1 %)	163 (1,7 %)	1 (0,0 %)	580 (6,1 %)	62 (0,7 %)	9462
*N*	5061 (76,5 %)	62 (0,9 %)	1345 (20,3 %)	38 (0,6 %)	19 (0,3 %)	4 (0,1 %)	146 (2,2 %)	1 (0,0 %)	6614
*O*	9622 (81,7 %)	894 (7,6 %)	1772 (15,1 %)	105 (0,9 %)	150 (1,3 %)	4 (0,0 %)	99 (0,8 %)	22 (0,2 %)	11.774
*P*	2403 (63,1 %)	178 (4,7 %)	1066 (28,0 %)	217 (5,7 %)	3 (0,1 %)	13 (0,3 %)	90 (2,4 %)	18 (0,5 %)	3810
*Md* *IQR*	*67,6* *%* *(63,0–74,0)*	*3,2* *%* *(1,7–3,8)*	*21,5* *%* *(16,4–26,4)*	*2,0* *%* *(1,5–3,1)*	*0,2* *%* *(0,1–1,5)*	*0,4* *%* *(0,1–2,1)*	*1,6* *%* *(1,1–2,5)*	*0,7* *%* *(0,4–5,2)*	–

*Md* Median, *IQR* Interquartilabstand, *n.* *n.* *b.* nicht näher bezeichnet

*ICD J13, J14, J15, J16 und J18

Die Betrachtung der Verteilung der AB-Verordnungen der Praxen über die Quartale ergab: Auf das jeweils erste Quartal – über alle Erhebungsjahre 2015–2018 – entfielen ca. 25–35 %, auf das zweite ca. 20–25 %, im dritten lag das Gros bei ca. 15–20 % und im vierten bei ca. 20–25 % (Tab. 4).

**Tab. 4:** Übersicht über alle 1:1-verknüpften Fälle (bezogen auf Diagnosen/Verordnungen) nach Praxis

	Quartal über alle Jahre				Gesamt
	Q1	Q2	Q3	Q4	
*A*	277 (34,0 %)	195 (24,0 %)	171 (21,0 %)	171 (21,0 %)	814
*B*	360 (35,7 %)	235 (23,3 %)	188 (18,6 %)	226 (22,4 %)	1009
*C*	17 (25,8 %)	15 (22,7 %)	22 (33,3 %)	12 (18,2 %)	66
*D*	278 (27,5 %)	230 (22,8 %)	224 (22,2 %)	278 (27,5 %)	1010
*E*	159 (32,8 %)	122 (25,2 %)	94 (19,4 %)	110 (22,7 %)	485
*F*	122 (37,2 %)	69 (21,0 %)	56 (17,1 %)	81 (24,7 %)	328
*G*	153 (30,7 %)	131 (26,3 %)	106 (21,3 %)	108 (21,7 %)	498
*H*	141 (35,3 %)	105 (26,3 %)	69 (17,3 %)	84 (21,1 %)	399
*I*	155 (25,0 %)	168 (27,1 %)	126 (20,3 %)	171 (27,6 %)	620
*J*	41 (40,6 %)	19 (18,8 %)	20 (19,8 %)	21 (20,8 %)	101
*K*	97 (28,0 %)	87 (25,1 %)	70 (20,2 %)	93 (26,8 %)	347
*L*	76 (32,1 %)	55 (23,2 %)	31 (13,1 %)	75 (31,6 %)	237
*M*	563 (30,3 %)	477 (25,7 %)	373 (20,1 %)	446 (24,0 %)	1859
*N*	239 (31,6 %)	179 (23,7 %)	149 (19,7 %)	189 (25,0 %)	756
*O*	656 (34,0 %)	451 (23,4 %)	323 (16,8 %)	497 (25,8 %)	1927
*P*	203 (30,1 %)	157 (23,3 %)	136 (20,1 %)	179 (26,5 %)	675
*Md* *IQR*	*31,9* *%* *(29,6–34,3)*	*23,6* *%* *(23,1–25,3)*	*20,0* *%* *(18,3–20,5)*	*24,4* *%* *(21,6–26,6)*	–

*Md* Median, *IQR* Interquartilabstand

Sodann erfolgte eine Analyse der Korrelation von AB-Verordnungen und „einschlägigen“ Infektionsdiagnosen. Dazu wurden zunächst aus der Gesamtzahl der Diagnosen (*n* = 90.044) diejenigen Fälle selektiert, bei denen zum jeweiligen Patienten nur eine solche Diagnose vorlag (*n* = 41.078). Analog wurden bei den Verordnungen aus der Gesamtzahl von 28.248 die jeweils ersten pro Quartal selektiert (*n* = 24.404); ausgeschlossen wurden 3844 Verordnungen. Letztlich konnten 41.078 Diagnosen 11.131 Verschreibungen zugeordnet werden (Abb. Z2).

**Abb. 1:**
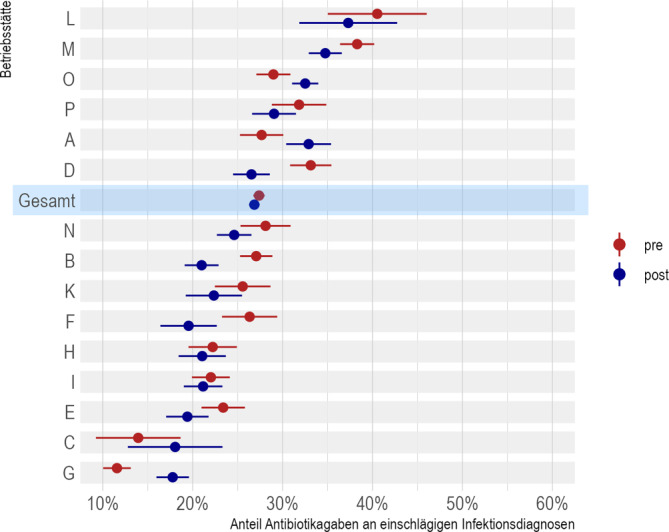
Anteil Antibiotikaverordnungen an einschlägigen Infektionsdiagnosen über den gesamten Studienzeitraum, vor und nach Start des AnTiB-Projektes, aufsteigend nach dem jeweiligen Praxismittelwert (mit 95 %-KI). (Quelle: eigene Abbildung)

Schließlich wurden die AB-Verordnungen bei „einschlägigen“ Diagnosen differenziert in den Zeitabschnitt vor Start des AnTiB-Projektes am 01.01.2017 („prä“) bzw. danach („post“). Dabei zeigt sich, dass bei 11 der 16 Praxen der Anteil „post“ unter dem von „prä“ lag, bei 4 Praxen hingegen lag „post“ über „prä“ (Praxis J war „prä“ noch nicht aktiv). Während die Unterschiede prä/post in den einzelnen Praxen zum Teil deutlich ausfallen, fällt der Unterschied gemittelt über alle Praxen nur gering aus (Abb. 1). Dabei zeigt sich eine Tendenz zur Homogenität der Verordnungsraten; betrug deren Streuung prä-AnTiB noch zwischen 12–41 %, fiel sie post-AnTiB mit 18–37 % zum einen geringer aus und lag zudem insgesamt – wenngleich gering – unter dem Prä-AnTiB-Wert.

Werden die Penicilline der Subgruppen „beta-Lactamase-sensitiv“ (ATC J01CE) bzw. „…-resistent“ (ATC J01CF) als „Schmalspektrum-AB“ gewertet und allen übrigen AB – als „Breitspektrum-AB“ – gegenübergestellt, so erfolgten post-AnTiB mit *n* = 1491 (25,4 %) von insgesamt 5870 AB-Verordnungen deutlich mehr an „Schmalspektrum-AB“-Verordnungen vs. prä-AnTiB mit *n* = 879 (16,7 %) von 5261.

### Ergebnisse der Regressionsmodelle.

Tab. 5 zeigt die Ergebnisse des gemischten Poisson-Modells für die Anzahl an verschriebenen AB über den gesamten Studienzeitraum, adjustiert für die Anzahl an AMP sowie Alter und Geschlecht. Für die Exposition des Quartals ergibt sich ein relatives Risiko von 0,98 (95 %-KI: 0,98;0,98, *p*-Wert < 0,0001), welches eine quartalsweise Reduktion der verschriebenen AB um im Mittel 2 % impliziert.

**Tab. 5:** Ergebnisse des gemischten Poisson-Modells, Outcome: Anzahl der verschriebenen Antibiotika (adjustiert für Anzahl an Arzneimittelpatient:innen)

Kovariable	Relatives Risiko	95 %-Konfidenzintervall	*p*-Wert
Geschlecht (weiblich vs. männlich)	1,10	[1,07; 1,12]	< 0,0001
Quartal	0,98	[0,98; 0,98]	< 0,0001
Alter 2–< 6 vs. 0–< 2	2,21	[2,12; 2,29]	< 0,0001
Alter 6–< 10 vs. 0–< 2	2,01	[1,93; 2,09]	< 0,0001
Alter 10–< 15 vs. 0–< 2	1,66	[1,59; 1,74]	< 0,0001
Alter 15–< 18 vs. 0–< 2	2,42	[2,28; 2,56]	< 0,0001

*Kovariablen:* Altersgruppe, Geschlecht, Quartal; *zufällige Effekte:* Betriebsstättennummer (BSNR); *Akaike Information Criterion (AIC):* 12.839,43; *Bayesian Information Criterion (BIC):* 12.845,61

(vgl. weitere Informationen im Onlinematerial)

Tab. Z3a und Z3b im Online-Supplement enthalten die Ergebnisse der gemischten Poisson-Modelle mit der Anzahl an verschriebenen AB als Outcome, adjustiert für die Anzahl an antibiotikarelevanten Diagnosen und Diagnosegruppen, jeweils getrennt für den Zeitraum Prä- und Post-AnTiB. Für die Exposition des Quartals ergibt sich im Prä-AnTiB-Zeitraum ein relatives Risiko von 0,98 (95 %-KI: 0,98; 0,98, *p*-Wert < 0,0001), im Post-AnTiB-Zeitraum von 0,99 (95 %-KI: 0,99; 0,99, *p*-Wert < 0,0001). In beiden Zeiträumen gibt es demnach Evidenz für eine quartalsweise Verringerung der verschriebenen AB um jeweils 2 % bzw. 1 %. Es ergibt sich weiterhin eine Standardabweichung der verschriebenen AB über alle Arztpraxen hinweg von 0,371 im Prä-AnTiB- und 0,299 im Post-AnTiB-Zeitraum (Standardabweichung des zufälligen Intercept). Diese zeitliche Verringerung der Varianz deutet auf eine einheitlichere „Verschreibekultur“ im Post-AnTiB-Zeitraum hin.

Tab. Z4 zeigt die Ergebnisse der Sensitivitätsanalyse. Für den Einfluss des Zeitraums ergibt sich ein relatives Risiko von 0,95 (95 %-KI: 0,91; 0,98, *p*-Wert < 0,0001), sodass die Analyse eine im Mittel abnehmende Anzahl an verordneten AB im Post-AnTiB- verglichen mit dem Prä-AnTiB-Zeitraum bestätigt.

## Diskussion

In dieser Arbeit wurde angestrebt, das AB-Verordnungsverhalten der Kinderarztpraxen im KV-Bezirk Bielefeld näher zu analysieren und dabei die Faktoren Alter, Geschlecht, Verordnungshäufigkeit der jeweiligen Praxen sowie Zuordnung der Verordnungen zu bestimmten Infektionsdiagnosen zu berücksichtigen.

Ausgewertet werden konnten 28.248 AB-Verordnungen, welche 262.969 Arzneimittelpatienten (AMP) gegenüberstehen. Diese konnten auf 90.044 Infektionsdiagnosen bezogen werden. Daraus ließen sich letztlich 11.131 1:1-Verknüpfungen für eine diagnosebezogene Auswertung generieren. Mit Blick auf das Verordnungsverhalten der einzelnen Praxen fanden sich, adjustiert auf die Nennergröße AMP, trotz vergleichbarer Alters- und Geschlechtsstruktur z. T. deutliche Unterschiede. Dies betraf sowohl die quantitative Verordnungshäufigkeit als auch die qualitative Zusammensetzung der verordneten AB-Substanzgruppen.

### Methodische Diskussion

Die Einteilung der Altersgruppen, ebenso bei [13], passt gut zu der Dynamik der altersspezifischen Verordnungsraten mit einem steilen Anstieg in den ersten Lebensjahren, gefolgt von einem ebenso steilen Abfall bis etwa 12 Jahre und dann wieder einem Anstieg [2]. Zu beachten ist allerdings eine Unschärfe der AMP-Altersangaben, da diese lediglich Geburtsjahre enthalten, was sich insbesondere bei Altersberechnungen in „0–< 2 Jahre“ auswirkt.

Zwischen den Praxen zeigte sich überwiegend ein ausgeglichenes Geschlechterverhältnis. Eine geschlechtsbezogene Analyse der Verordnungshäufigkeiten wurde nur für die Altersgruppe 15 bis < 18 durchgeführt, da dort größere Differenzen zu erwarten waren [2]. Hierbei zeigte sich auch bei uns eine mit 57 % bei den Mädchen gegenüber 43 % bei den Jungen höhere Verordnungsrate, bei einem geringfügigen Überwiegen der Jungen (52 % vs. 48 %). Eine weitere Studie untersuchte die wiederholte Verordnung einer Substanzgruppe innerhalb eines Jahres. Dabei fanden sich systemische AB (J01) bei Jungen und Mädchen praktisch aller Altersstufen unter den beiden am häufigsten verordneten Medikamenten (Ausnahme: Jungen 6–12 Jahre, Platz 3). In der Altersgruppe 13–17 lag die Prävalenz bei den Mädchen mit 35,2, verglichen mit den Jungen mit 18,8 – praktisch doppelt so hoch – jeweils bezogen auf 1000 Personenjahre [14].

Die AB wurden nach Substanzgruppen unterteilt, jedoch nicht nach Einzelsubstanzen differenziert, mit Ausnahme der „Schmalspur-Penicilline“, was bei künftigen Auswertungen noch weiter abgestuft werden könnte. Nicht erfasst wurden Antibiotika zur topischen Anwendung, insbes. Augentropfen, die ebenfalls zum Resistenzgeschehen beitragen.

Die Auswahl der Infektionsdiagnosen folgte der Vorlage von [8], ergänzt um die „unspezifischen“ ICD-Codes A49 und B99 sowie um dermatologische Codes. Trotz dieses Umfangs blieben einzelne Diagnosen unberücksichtigt, die ebenfalls eine AB-Verordnung indizieren könnten. Es wäre zu überlegen, inwieweit an dieser Stelle auch Ausschlussdiagnosen zum Einsatz kommen könnten [15].

Ideal wäre eine unmittelbare Verknüpfung von Diagnose- und Verordnungsdaten, was im derzeitigen Dokumentationssystem jedoch nicht verfügbar ist. Hilfsweise erfolgte daher ein 1:1-Matching von AB-Verordnungen im jeweiligen Quartal. Es ist offensichtlich, dass dadurch eine deutliche Restriktion der an sich verfügbaren Daten resultiert, in unseren Ergebnissen auf 39,4 % (11.131 von 28.248 verknüpfbaren Verordnungen). Dies wird allerdings dadurch kompensiert, dass zumindest in diesen Fällen das „Hintergrundrauschen“ verschiedener Infektionsdiagnosen innerhalb eines Quartals ausgeblendet wird, wodurch Verordnungsvarianzen bei chronischen Erkrankungen besser sichtbar werden (z. B. [16]).

Fälle mit „einer AB-Verordnung zu *n* > 1 Infektionsdiagnosen“ pro Quartal wurden exkludiert, hingegen Fälle mit „einer Infektionsdiagnose zu *n* > 1 AB-Verordnungen“ integriert, dann mit Einbeziehung des ersten im Quartal verordneten AB unter der Vorstellung, dass weitere Verordnungen auf einen unzureichenden primären Behandlungserfolg bzw. ein Wiederaufflammen der Infektion hin erfolgten. Dies könnte dort zu Fehlzuordnungen geführt haben, wo Diagnosecodierungen bei bestimmten AB-Verordnungen unterlassen wurden. Der zahlenmäßige Einfluss solcher Fälle wird zwar als gering eingeschätzt, jedoch bleibt die Verknüpfung von Diagnose- und Verordnungsdaten aufgrund der unterschiedlichen Zeitraster immer eine Abwägung zwischen der Erfassung möglichst vieler Fälle und dabei potenziell größer werdenden Störquellen.

AMP-Zahlen als „Nennergröße“ werden in der Versorgungsforschung oft eingesetzt [17]. Sie liefern allerdings nur Näherungswerte des Verordnungsverhaltens einzelner Ärzt:innen bzw. Praxen bzw. bergen die Möglichkeit von Verzerrungen: Verordnen Praxen insgesamt wenige Arzneimittel, können an sich rational verordnete AB-Mengen überproportional hoch aussehen und umgekehrt niedrig in Praxen, die ein hohes allgemeines Verschreibungsverhalten pflegen. Des Weiteren ist zu bedenken, dass als Besonderheit in der Pädiatrie bis zum Alter von 12 Jahren auch nichtverschreibungspflichtige Arzneimittel verordnungsfähig sind. Daher ist für unter 12-Jährige das Spektrum an verordneten Arzneimitteln deutlich umfangreicher und folglich die AMP-Anzahl auch entsprechend größer als bei über 12-Jährigen, was Vergleiche über diese Altersgrenze hinweg nicht erlaubt.

Das Aufzeigen von AB-Verordnungsvarianzen innerhalb einer Arztgruppe kann als Ansatzpunkt zur Motivation hin zu einem niedrigeren Verordnungslevel dienen. Dies zeigt sich naturgemäß am deutlichsten auf der Ebene einzelner Ärzt:innen (LANR) bzw. wird durch die Aggregierung auf der Ebene von Praxen (BSNR) verwischt. Offen bleibt, inwieweit das Verordnungsverhalten einer Praxis in sich homogen ist, wobei auch die von einzelnen Ärzt:innen versorgte Klientel mit ggf. unterschiedlichen Morbiditäten zu berücksichtigen ist.

In diesem Kontext wäre noch interessant, ob niedrige AB-Verordnungsraten einzelner Praxen mit einer höheren stationären Einweisungsquote assoziiert sind, womit eine AB-Verordnung in den stationären Bereich verlagert werden könnte. Stationär sind individuelle Diagnosedaten, umgekehrt zu ambulant, qua DRG-System^5^ recht hoch aufgelöst und zeitlich (auf den i. d. R. kurzen stationären Aufenthalt) fokussiert verfügbar, jedoch stehen dort AB-Verordnungsdaten oft nicht patientenindividuell zur Verfügung. Diese Datenquellen zu vereinheitlichen bzw. zusammenzuführen, wäre eine lohnenswerte Aufgabe für ABS!

### Inhaltliche Diskussion

Ein direkter Vergleich unserer Ergebnisse mit anderen Studien aus Deutschland ist aus methodischen Gründen nicht ohne Weiteres möglich. Im Projekt RESIST [15], Laufzeit 2016–2020, wurde ein komplexer Ansatz verfolgt, der das AB-Verordnungsverhalten verschiedener ambulanter FG im Kontext mit einer multimodalen Intervention untersuchte. Ein Outcome war die AB-Verordnungsrate der Praxen vor und nach Intervention sowohl über alle Diagnosen als auch selektiv bei Atemwegsinfekten. Bezogen explizit auf die Kinderärzt:innen lag die generelle Verordnungsrate prä bei 9,7 % und post bei 8,3 % und die atemwegsbezogene prä bei 17,8 % und post bei 14,4 %. Ähnlich zu uns waren in den pädiatrischen Altersgruppen nach Adjustierungen prä-/postinterventionell kaum Unterschiede der Raten zu verzeichnen. Eine große französische Studie analysierte 2015–2017 ebenfalls das ambulante AB-Verordnungsverhalten bei Kindern [18]. Pädiater:innen verordneten dabei AB in 21,6 % aller Konsultationen. Internationale Vergleiche sind allerdings problematisch v. a. aufgrund unterschiedlicher Versorgungssysteme etc.

#### Korrelation zu saisonalen respiratorischen Infekten.

AB-Verordnungen werden auch stark von der Epidemiologie bzw. Saisonalität von Atemwegsinfekten beeinflusst. Obwohl diese weit überwiegend viral bedingt sind und somit keine Indikation für einen AB-Einsatz darstellen, lösen sie durch Schwierigkeiten in der Abgrenzung zu bakteriellen Erkrankungen oft AB-Verordnungen aus. In der Kinderheilkunde betrifft dies insbesondere Influenza A und B sowie RSV. Deren Saisonalität erklärt die Schwankungen der Verordnungsraten über die Quartale mit i. d. R. einem Maximum in Q1, gefolgt von Q4, Q2 und Q3 mit den jeweils niedrigsten Raten. Allerdings variiert dies z. T. sehr deutlich, weswegen jahreszeitliche bzw. jahresübergreifende Trends von AB-Verordnungsraten sowohl die jeweilige Epidemiologie als auch Veränderungen im Verordnungsverhalten widerspiegeln können. Im Kontext dieser Arbeit erscheint relevant, dass die Influenzasaison 2017/2018 vom Robert Koch-Institut (RKI) als „ungewöhnlich stark“ eingeschätzt wurde – mit u. a. vermehrten Arztkonsultationen und Hospitalisationen. In der Pädiatrie versursacht Influenza z. B. virale Otitiden und Pneumonien, die sich nicht sicher von einer bakteriellen Sekundärinfektion abgrenzen lassen, mit Anlass sowohl für nichtindizierte wie auch indizierte AB-Verordnungen. Die Saison 2018/2019 hingegen verlief „schwächer“ [19], was die vergleichsweise geringe Zunahme an AB-Verordnungen im 4. Quartal 2018 erklären könnte.

#### Rolle von Dauerdiagnosen.

Patient:innen mit bestimmten Dauerdiagnosen, hervorzuheben die zystische Fibrose (ICD E84), aber auch Uropathien (ICD N13) und Trisomie 21 (ICD Q90), können eine erhöhte AB-Verordnungshäufigkeit bedingen und damit zu Verzerrungen führen. Wir haben unseren Datensatz auf diese Diagnosen überprüft und in den jeweiligen Praxen nur einzelne solcher Fälle gesehen, die keinen relevanten Einfluss auf die Verordnungshäufigkeit hatten.

#### Rolle von AB-, insbes. Penicillin-Allergien.

Im gesamten Diagnosedatensatz mit ca. 500.000 Diagnosen gab es – etwas überraschend – nur je einmal die Angabe einer Allergie auf Penicillin bzw. auf ein anderes AB! Eine Einbeziehung solcher Allergiediagnosen wäre interessant bei der Frage, inwieweit die Auswahl bestimmter ggf. Reserve-AB auch von anamnestischen Allergien mitbeeinflusst wird. Hierbei wäre zunächst denkbar, dass anstelle des spezifischen Codes einer AB-Allergie nur unspezifisch „Allergie“ kodiert wurde. Hinweise aus der Praxis besagen jedoch, dass solche Allergien zwar in der „Cave-Rubrik“ einer Patientenakte vermerkt werden, offensichtlich aber nicht in der an die KV übermittelten Diagnosenliste. Das sachgerechte Identifizieren von AB-Allergien bzw. das „De-labeling“ ggf. unzutreffender Allergiezuordnungen [20] wäre eine lohnende ABS-Aufgabe (nicht nur) in der Pädiatrie.

#### Zuverlässigkeit von Diagnosekodierungen.

Unklar ist, wie zuverlässig – nach vergleichbaren Kriterien und möglichst differenziert – die hier untersuchten Diagnosen kodiert wurden. Bei uns reicht der Anteil unspezifischer B99-Diagnosen – bei einem Median um 1 % – bis 26,5 %, was auf ein eingeschränktes Verständnis einer guten Kodierqualität hinweisen könnte. Selbst bei der spezifischen Diagnose „Scharlach“ findet sich eine Spannweite von 1–6 %, was durch unterschiedlich angewandte Diagnosekriterien oder auch unterschiedlichen Einsatz von (Streptokokken‑)Abstrichen zu erklären wäre. Eine Erklärung wäre, dass eine gute Kodierqualität vom Abrechnungssystem nicht unbedingt honoriert wird bzw. deren Sinn auch „medizinisch“ nicht eingesehen wird. Dies schränkt die Aussagekraft unserer Analysen im ambulanten Sektor ein – im Unterschied zum stationären Sektor, wo Diagnosen qua DRG-System immer differenzierter gestellt werden und dort für diagnosebezogene Auswertungen geeigneter erscheinen.

Mit dem offiziellen Inkrafttreten der pädiatrischen AB-Verordnungsempfehlungen zum 01.01.2017 als markantem Stichzeitpunkt sind die Unterschiede prä/post nur gering und wären auch im Rahmen eines allgemein rückläufigen AB-Verordnungsverhaltens zu interpretieren. Allerdings ist die Veränderung von „Verordnungskulturen“ (aktuelle Ansätze z. B. bei [21]) eine langfristig angelegte Strategie, deren Effekte sich noch nicht in so kurzen Zeitabschnitten zeigen. Eine längerfristige Auswertung war bislang nicht möglich, da ab 2020 die COVID-19-Pandemie das Infektions- und Verordnungsgeschehen tiefgreifend verändert hat.

Nach Jahren des anhaltenden Rückgangs von AB-Verordnungen [22–24] deutet sich aktuell eine Trendumkehr an: So lagen die Verordnungen in Westfalen-Lippe (GKV, alle Altersgruppen) im 1. Halbjahr 2023 mit 1,728 Mio. wieder auf dem Niveau des 1. Halbjahres 2019, nach einem historischen COVID-19-bedingten Tiefpunkt mit 857.000 im 1. Halbjahr 2021 [25].

Insgesamt ist es eine komplexe Aufgabe, im ambulanten Sektor das arztindividuelle bzw. praxisbezogene AB-Verordnungsverhalten in eine Beziehung einerseits zu einer Nennergröße (AMP) und andererseits zur Morbidität der jeweils versorgten Klientel zu setzen. Dennoch sind solche Anstrengungen unabdingbar und sollten auch auf andere Medikamentengruppen bzw. andere medizinische Fachbereiche übertragen werden. Hierfür bietet die vorliegende Arbeit entsprechende Forschungsansätze.

## Fazit

Varianzen im AB-Verordnungsverhalten sowohl auf regionaler Ebene als auch innerhalb lokaler Fachgruppen lassen sich nur zum Teil durch unterschiedliche Zusammensetzung bzw. Morbidität der versorgten Klientel zurückführen. Zur tiefergehenden Analyse erscheint eine Verknüpfung von Verordnungen und Infektionsdiagnosen unabdingbar. Der vorgestellte Ansatz ermöglicht dies und ist auch auf andere ambulante Fachgruppen anwendbar.

### Supplementary Information

Im Supplement finden sich Übersichten zu: den für die Analyse berücksichtigten ICD-10-Diagnosen, den Geschlechterverhältnissen in den einzelnen Praxen, den Modellberechnungen, den ein- bzw. ausgeschlossenen Fällen sowie zur Verordnungsheterogenität in den Praxen

## Footnotes

1 Anatomical Therapeutic Chemical Classification der Weltgesundheitsorganisation (WHO).

2 Lebenslange Arztnummer.

3 Betriebsstättennummer.

4 International Statistical Classification of Diseases and Related Health Problems, German Modification.

5 Diagnosis Related Groups, Klassifikationssystem für ein pauschaliertes Abrechnungsverfahren.
