# Supplementary material for: Analysis of factors influencing outpatient paediatric antibiotic prescriptions in Bielefeld from 2015 to 2018
Source: Bundesgesundheitsblatt Gesundheitsforschung Gesundheitsschutz. 2024 Jun 5;67(9):1010–20. [Article in German] doi: 10.1007/s00103-024-03891-9 (PMC11349860; doi:10.1007/s00103-024-03891-9)
Supplement: Supplementary file 1 — Im Supplement finden sich Übersichten zu: den für die Analyse berücksichtigten ICD-10-Diagnosen, den Geschlechterverhältnissen in den einzelnen Praxen, den Modellberechnungen, den ein- bzw. ausgeschlossenen Fällen sowie zur Verordnungsheterogenität in den Praxen [file 103_2024_3891_MOESM1_ESM.pdf]

Online-Material zu *Bornemann et al. (2024):*
„Analyse von Einflussfaktoren auf ambulante pädiatrische Antibiotikaverordnungen in Bielefeld 2015–2018“

Ergänzende Informationen zu Tab. 5:

Beispiel Interpretation Quartal: Mit zunehmendem Quartal sinkt die Wahrscheinlichkeit für Antibiotikaverschreibungen bzw. die Anzahl an Verschreibungen nimmt im Mittel von einem Quartal zum nächsten um 2% ab.
Visualisierung der Prädiktion (für „mittlere“ Studienteilnehmer: weiblich, Altersgruppe 10 bis <15, AMP 117):

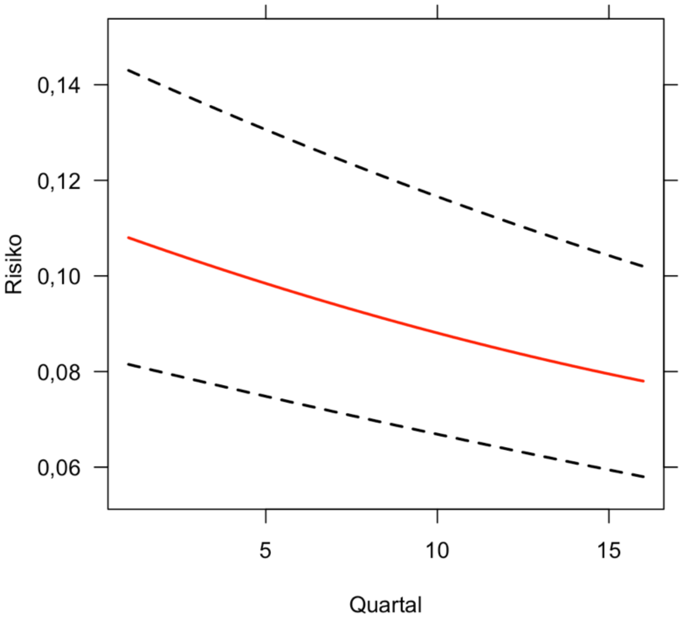

(Quelle: Eigene Abbildung).

Zusätzliche Tabellen

Tab. Z1: In dieser Arbeit berücksichtigte ICD-10-Diagnosen im Vergleich zu Schulz et al. 2014

ICD-Code	Schulz 2014	diese Arbeit	ICD-Klartext
Kap. I – Bestimmte infektiöse und parasitäre Krankheiten			
A38	+	+	Scharlach
A49	–	+	Bakterielle Infektionen n. n. bez.
B99	–	+	Sonstige Infektionskrankheiten
Kap. VIII – Krankheiten des Ohres und des Warzenfortsatzes			
H65	+	+	Nichteitrige Otitis media
H66	+	+	Eitrige und n. n. bez. Otitis media
Kap. X – Krankheiten des Atmungssystems			
J00	+	+	Akute Rhinopharyngitis (Erkältungsschnupfen)
J01	+	+	Akute Sinusitis
J02	+	+	Akute Pharyngitis
J03	+	+	Akute Tonsillitis
J04	+	+	Akute Laryngitis und Tracheitis
J06	+	+	Akute Inf. an mehreren oder n. n. bez. Lok. d. ob. Atemwege
J13	+	+	Pneumonie durch Streptococcus pneumoniae
J14	+	+	Pneumonie durch Haemophilus influenzae
J15	+	+	Pneumonie durch Bakterien, anderenorts nicht klassifiziert
J16	+	+	Pneumonie durch sonst. Infektionserreger, anderenorts nicht klassifiziert
J18	+	+	Pneumonie, Erreger n. n. bez.
J20	+	+	Akute Bronchitis
J21	+	+	Akute Bronchiolitis
J22	+	+	Akute Infektion der unteren Atemwege, n. n. bez.
Kap. XII – Krankheiten der Haut und der Unterhaut			
L01	–	+	Impetigo
L02	–	+	Hautabszess
L03	–	+	Phlegmone
L08	–	+	Sonstige lokale Inf. der Haut und Unterhaut (Pyodermie ...)
Kap. XIV – Krankheiten des Urogenitalsystems			
N30	+	+	Zystitis
N39.0	+	+	Harnwegsinfektion, Lokalisation n. n. bez.

Tab. Z2: (Referenz: Tab. 1) Altersgruppe 15 – <18 pro Praxis – Geschlechterverhältnis jeweils bei AMP insges. und bei-Antibiotikaverordnungen

Praxis	n AMP m	n AMP w	% AMP m	% AMP w	n AB m	n AB w	% AB m	% AB w
A	334	336	49,9	50,1	58	88	39,7	60,3
B	318	301	51,4	48,6	23	49	31,9	68,1
C	84	81	50,9	49,1	1	12	7,7	92,3
D	1.767	1.575	52,9	47,1	108	166	39,4	60,6
E	437	360	54,8	45,2	24	57	29,6	70,4
F	87	102	46,0	54,0	4	33	10,8	89,2
G	480	416	53,6	46,4	59	58	50,4	49,6
H	109	204	34,8	65,2	21	54	28,0	72,0
I	249	258	49,1	50,9	51	59	46,4	53,6
J	39	52	42,9	57,1	5	11	31,3	68,8
K	214	169	55,9	44,1	9	21	30,0	70,0
L	148	92	61,7	38,3	15	10	60,0	40,0
M	791	609	56,5	43,5	146	108	57,5	42,5
N	143	164	46,6	53,4	37	54	40,7	59,3
O	593	562	51,3	48,7	118	124	48,8	51,2
P	444	536	45,3	54,7	35	46	43,2	56,8
gesamt	6.237	5.817	51,7	48,3	714	950	42,9	57,1

Tab. Z3a: Ergebnisse des gemischten Poisson-Modells, Outcome: Anzahl der verschriebenen Antibiotika, die einer Diagnose zugeordnet werden können (Zeitraum prä-AnTiB)

Kovariable	Relatives Risiko	95%-Konfidenzintervall	p-Wert
**Quartal**	0,98	[0,98; 0,98]	< 0,0001
**Bakterielle Inf., n. n. bez. vs. Atmungssystem**	3,03	[2,95; 3,12]	<0,0001
**Haut vs. Atmungssystem**	2,66	[2,61; 2,71]	<0,0001
**Otitis vs. Atmungssystem**	2,55	[2,52; 2,57]	<0,0001
**Scharlach. vs. Atmungssystem**	4,44	[4,37; 4,51]	<0,0001
**Sonstige Infektionskrankheiten vs. Atmungssystem**	0,80	[0,76; 0,84]	<0,0001
**Urogenitalsystem vs. Atmungssystem**	2,93	[2,84; 3,02]	<0,0001

Tab. Z3b: Ergebnisse des gemischten Poisson-Modells, Outcome: Anzahl der verschriebenen Antibiotika, die einer Diagnose zugeordnet werden können (Zeitraum post-AnTiB)

Kovariable	Relatives Risiko	95%-Konfidenzintervall	p-Wert
**Quartal**	0,99	[0,99; 0,99]	< 0,0001
**Bakterielle Inf., n. n. bez. vs. Atmungssystem**	3,23	[3,13; 3,33]	<0,0001
**Haut vs. Atmungssystem**	2,88	[2,83; 2,93]	<0,0001
**Otitis vs. Atmungssystem**	2,58	[2,55; 2,60]	<0,0001
**Scharlach. vs. Atmungssystem**	4,90	[4,82; 4,98]	<0,0001
**Sonstige Infektionskrankheiten vs. Atmungssystem**	0,73	[0,70; 0,76]	<0,0001
**Urogenitalsystem vs. Atmungssystem**	4,62	[4,62; 4,93]	<0,0001

Tab. Z4: Ergebnisse des gemischten Poisson-Modells, Outcome: Anzahl der verschriebenen Antibiotika (beschränkt auf die Verschreibungen, die einer Diagnose zugeordnet werden können) im gesamten Zeitraum

Kovariable	Relatives Risiko	95%-Konfidenzintervall	p-Wert
**Zeitraum (post vs. prä)**	0,95	[0,91; 0,98]	< 0,0001
**Bakterielle Inf., n. n. bez. vs. Atmungssystem**	2,92	[2,55; 3,34]	0,0039
**Haut vs. Atmungssystem**	2,72	[2,50; 2,95]	<0,0001
**Otitis vs. Atmungssystem**	2,56	[2,45; 2,68]	<0,0001
**Scharlach. vs. Atmungssystem**	4,53	[4,22; 4,87]	<0,0001
**Sonstige Infektionskrankheiten vs. Atmungssystem**	0,74	[0,60; 0,91]	0,0049
**Urogenitalsystem vs. Atmungssystem**	3,42	[2,95; 3,98]	<0,0001

Zusätzliche Abbildungen

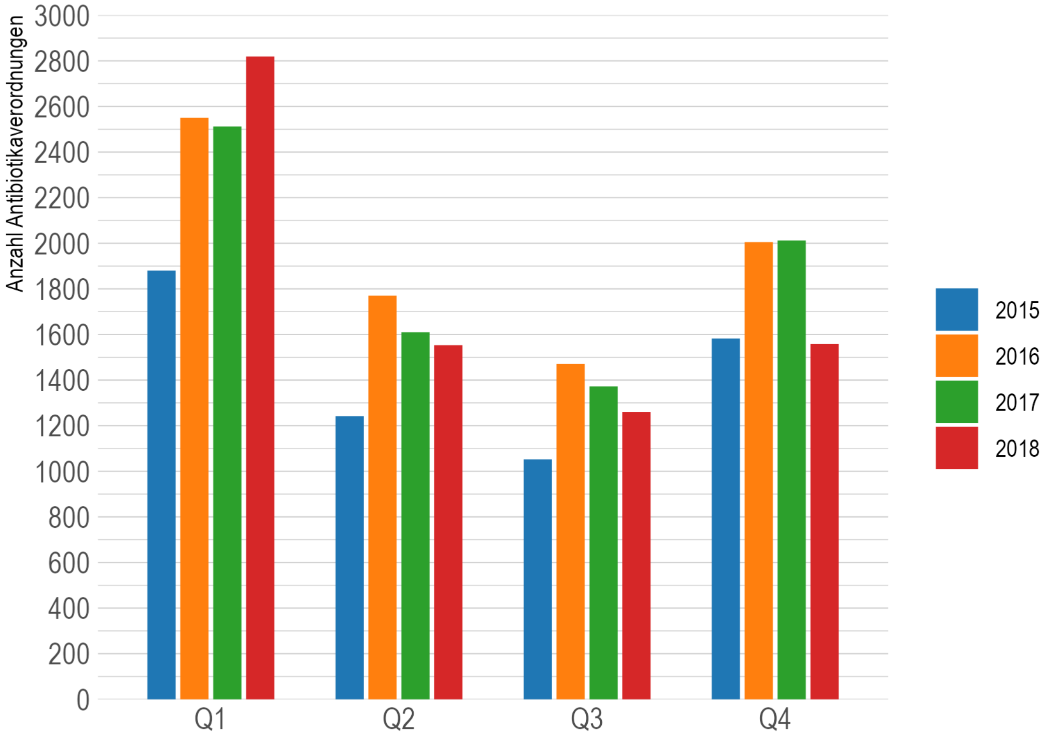

Abb. Z1: Anzahl Antibiotikaverordnungen pro Quartal im Untersuchungszeitraum 2015-2018 (Quelle: Eigene Abbildung).

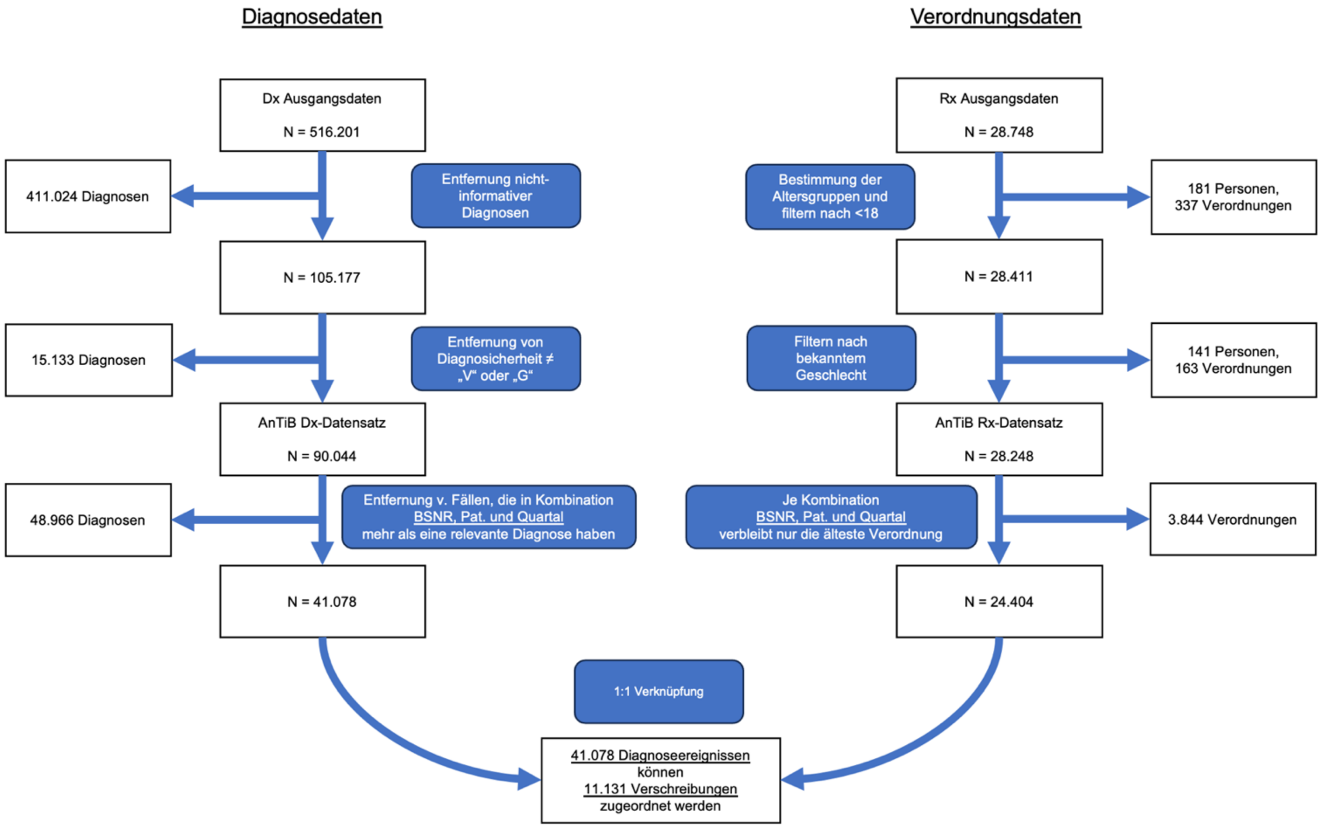

Abb. Z2: Flussdiagramm der ein- bzw. ausgeschlossenen Diagnosen und Antibiotikaverordnungen sowie deren algorithmisierter 1:1-Verknüpfung.
Aus der Gesamtzahl der einschlägigen Infektionsdiagnosen (n=90.044) wurden die singulären Diagnosen pro Quartal (n=41.078) eingeschlossen, hingegen wurden ausgeschlossen: Doppelnennungen 28.486, drei- 12.399, vier- 5.248, fünf- 1.615, sechs- 852, sieben- 126, acht- 152, zehn- 50, zwölf- 24 und vierzehnfache Nennungen 14 (zus. 48.966). Analog wurde aus der Gesamtzahl von Verordnungen (28.248) die singulären pro Quartal eingeschlossen (n=24.404), hingegen wurden ausgeschlossen 3.844 Verordnungen (darunter 176 Fälle, bei denen Patienten *am gleichen Tag* von derselben BSNR zwei unterschiedliche AB verordnet wurden, die folglich nicht über die Regel „älteste Verordnung im Quartal gilt" aufgelöst werden konnten). Zwischen den 41.078 Diagnosen und den 24.404 Verordnungen ließen sich 11.131 Verknüpfungen erstellen, die übrigen 13.273 Verordnungen erfolgten bei Patienten ohne singuläre Diagnose (Quelle: Eigene Abbildung).

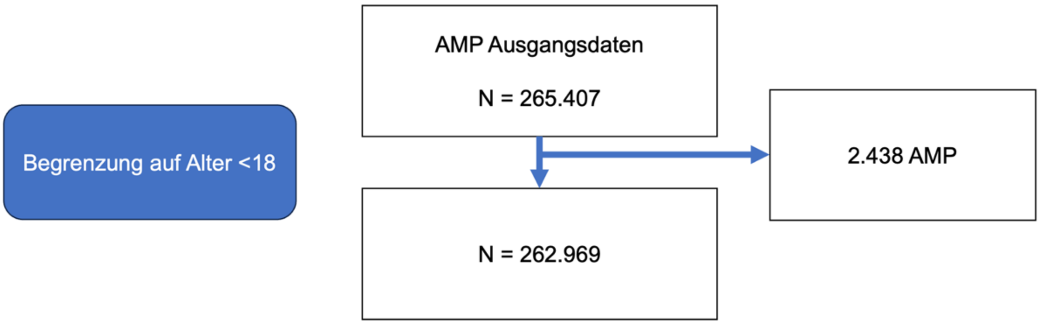

Abb. Z3: Flussdiagramm der ein- bzw. ausgeschlossenen AMP (Quelle: Eigene Abbildung).

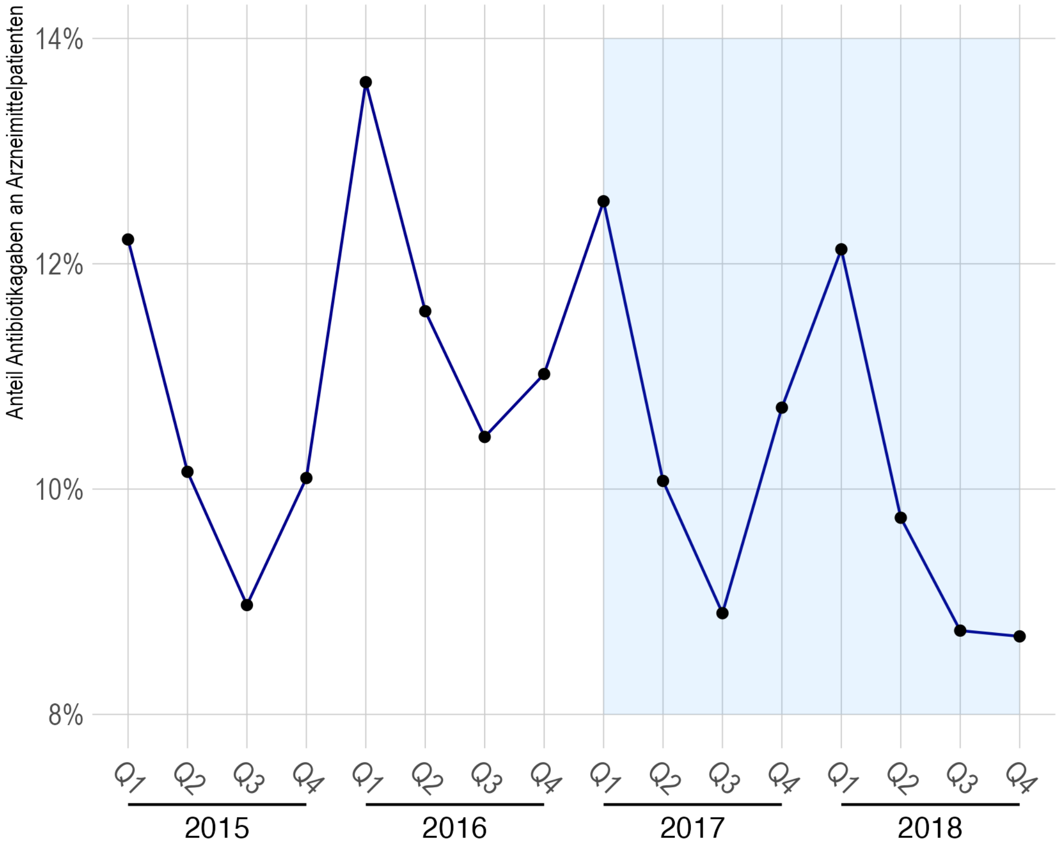

Abb. Z4: Anteile der Antibiotikaverordnungen an AMP im Verlauf der Studienquartale 2015-2018 gemittelt über alle 16 Praxen (Datenbasis: Tab. 1) (Quelle: Eigene Abbildung).

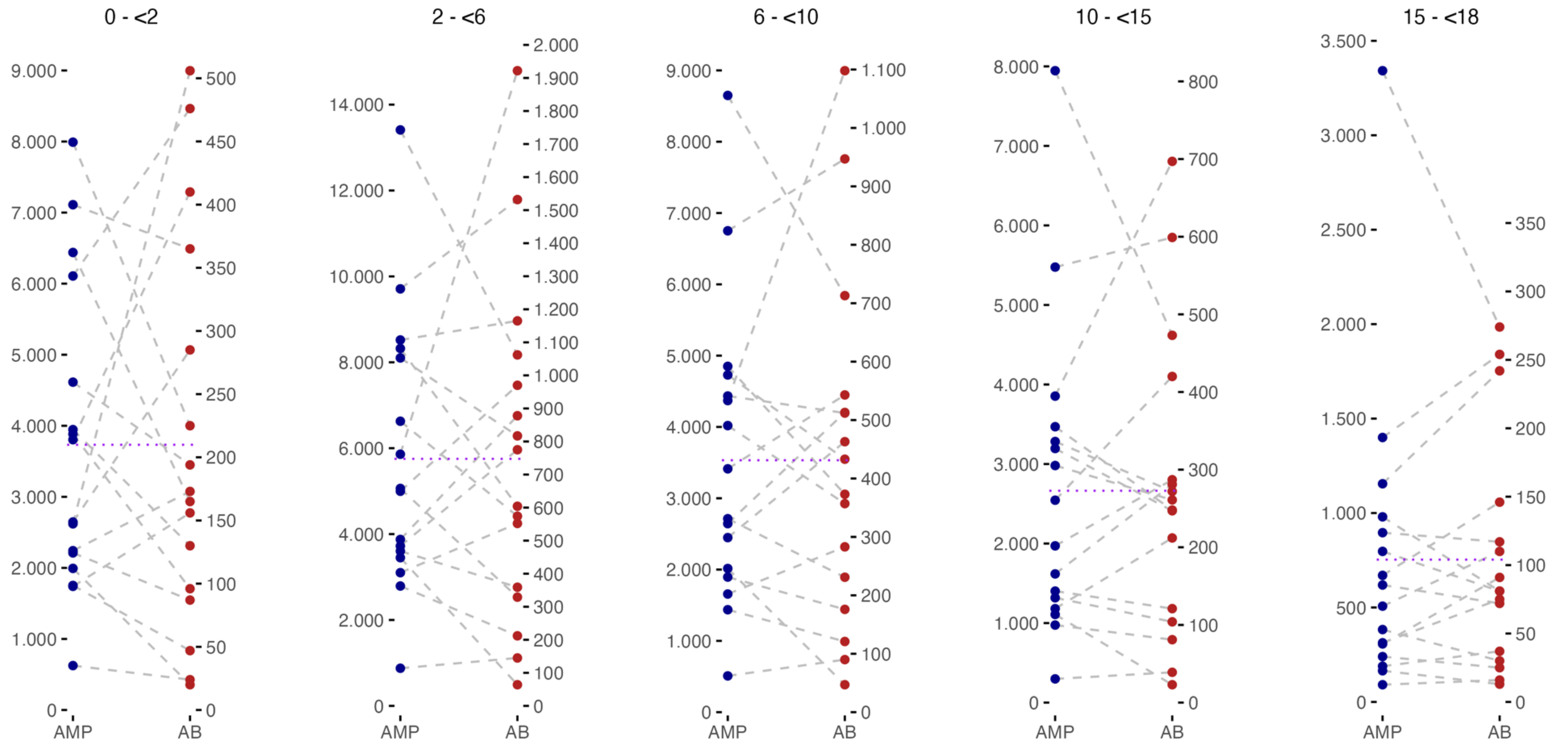

Abb. Z5: Heterogenität des Verhältnisses von AMP und Antibiotikaverordnungen der einzelnen Praxen nach Altersgruppen
Auf der jeweils linken Achse (blaue Punkte) sind die AMP-Zahlen je Praxis aufgetragen, rechts (rote Punkte) deren Anzahl an AB-Verordnungen. Die violette gepunktete Linie in jedem Graphen entspricht dem jeweiligen Mittelwert sowohl der AMP als auch der AB-Verordnungen. Am Beispiel der Altersgruppe 0-<2 bedeutet dies, dass auf die durchschnittlich 3.733 AMP 210 AB entfallen, also ein Verhältnis ungefähr 18:1. Eine Waagerechte bedeutet ein durchschnittliches, ein Graph mit positiver Steigung ein überdurchschnittliches Verordnungsverhalten. Nicht ohne weiteres zu werten ist der „Grad der Steigung" im Vergleich: Am Beispiel der Gruppe 0-<2 zeigt sich ausgehend vom obersten blauen Punkt mit einem Verhältnis von 225 AB / 8.000 AMP = 2,8% eine sehr eindrückliche „negative Steigung". Der blaue Punkt ziemlich genau auf der 2.000er-Marke hat nur 20 AB verordnet (= 1%), fällt aber flacher ab (Quelle: Eigene Abbildung).
